# Future Perspectives in Radiology: Artificial Intelligence for Responsible Imaging (AIRI)

**DOI:** 10.7759/cureus.80095

**Published:** 2025-03-05

**Authors:** Kunmilayo Olayeye, Christina Regine Owens-Charles, Jashkumar Choudhari, Esha Parikh, Zhengrong Jerome Liang

**Affiliations:** 1 Foundation, Nova Southeastern University Dr. Kiran C. Patel College of Osteopathic Medicine, Fort Lauderdale, USA; 2 Department of Radiology, Stony Brook University, Stony Brook, USA

**Keywords:** artifical intelligence, general radiology, guidelines in medicine, imaging modalities, machine learning in medicine, machine learning in radiology, machine learning (ml), radiation side effects, radiology and medical imaging, screening guidelines

## Abstract

Artificial intelligence (AI) has transformed the healthcare industry. In the field of radiology, AI has shown great promise in medical imaging, as it can enhance imaging accuracy, reduce diagnostic errors, and optimize workflow. However, the overuse and misuse of imaging is still a major ongoing problem, generating unnecessary costs, radiation exposure to patients, and delays in diagnosis. To address these challenges, we propose AI for responsible imaging (AIRI), a multimodal AI system to aid clinicians in making informed imaging decisions. AIRI is envisioned as a medical resource that would benefit the clinician, the radiologist, and, most importantly, the patient.

For clinicians, AIRI would process clinical data, such as labs, vitals, patient history and physical exam findings, and evidence-based appropriateness criteria, to determine if a medical image is clinically indicated based on the algorithmic likelihood that the imaging would provide necessary information for diagnosis. When imaging is indicated, AIRI would help the clinician determine logistic details such as what modality would give the most information for the radiologist to read, ordering details such as whether the use of contrast is indicated or not, and also how to best prepare the patient for what to expect to improve compliance and reduce the need for repeat scans. By assisting the non-radiologist clinician with these technical imaging decisions, AIRI has the potential to reduce unnecessary scans, minimize radiation exposure, and decrease healthcare costs.

For radiologists, AIRI with radiomic integration could provide preliminary interpretations for low-priority images or frequently seen cases, allowing them to focus more on high-acuity and complex cases. With radiomics, AIRI could detect subtle abnormalities and imaging patterns that may be overlooked by the human eye and interpret images distorted by artifacts, allowing for more diagnostic information to be retrieved from a single scan and reducing the need for a repeat. Additionally, AIRI is envisioned to help the radiologist triage cases and implement a fatigue detection protocol to help prevent burnout. AIRI for the radiologist would streamline workflow, improve diagnostic accuracy, reduce repeat scans, and alleviate the radiologist’s workload.

For the patient, all of the applications mentioned above would work to reduce exposure to excess and unneeded radiation and help reduce healthcare costs and time spent in the diagnostic stage. AIRI, with AI chatbot integration, may improve the patient experience by supplementing the physician’s explanation of imaging procedures and results, easing scan-related anxieties, giving personalized prep guidance, and finding imaging facilities with the most affordable imaging studies.

AIRI is a shift toward more responsible usage of medical imaging. In this editorial, we expand upon AIRI’s design, implementation, and its potential to mitigate areas of medical overuse in the field of radiology, as well as the existing benefits of AI in the medical industry and why we believe AIRI would be a promising addition in the field of medical innovation.

Appropriate use of imaging is essential because it alleviates costs by scaling back imaging services without compromising diagnostic integrity. We elaborate on this concept, early use cases, and the potential for AIRI to change the landscape of radiology and healthcare for clinicians, radiologists, and patients.

## Editorial

Introduction: context and motivation

It is no secret that the advancements in artificial intelligence (AI) have created a new domain in the healthcare industry. While integrating AI into health care is controversial, it has proven to be a practical resource for clinical advancement in the last few years. The contentious nature of AI in the healthcare industry is mainly due to fear of replacing human expertise. However, based on the ideas proposed by the generalist medical AI (GMAI) model, AI is not a replacement for healthcare professionals but a tool that promises enhanced diagnostic capabilities and improved patient outcomes [1]. Building upon the GMAI model, we propose expanding its application to radiology to develop a new system called AIRI, AI for responsible imaging. As a comprehensive tool designed to assist clinicians and radiologists, we envision that AIRI will allow for the more appropriate use of radiological imaging.

Radiology is one of the most significant elements of modern diagnostic medicine. Despite that, numerous concerns are linked to radiology because inappropriate imaging practices remain a considerable issue. These practices lead to cost, improper resource use, and adverse patient outcomes. The ultimate consequences of improper imaging studies are overexposure to radiation, delayed diagnosis due to the need for repeated imaging, and increased cost to the patient.

A study by Oren et al. discovered that in certain case scenarios, diagnostic testing is unnecessary and mostly yields incidentalomas, which complicate the focus of treatment [2]. They argue the need for well-orchestrated educational strategies and “rule-out” criteria to guide practitioners when ordering scans. Oren et al. discuss that the implementation of these educational strategies and rule-out criteria would help to address the financial and health consequences associated with unnecessary imaging [2]. However, “rule-out” criteria exist, but over-imaging remains a challenge. Examples of these criteria include the American College of Radiology (ACR) Appropriateness Criteria, the Canadian C-Spine Rule, the Ottawa Ankle Rules, and the Pediatric Emergency Care Applied Research Network (PECARN) Pediatric Head and C-Spine Computed Tomography (CT) Algorithm for appropriate use of imaging in specific clinical scenarios. An obvious issue with many of these guidelines is that their results can vary widely depending on the clinical context and the physician’s biases. In addition, these guidelines are often not individualized, and patient presentations frequently have subtleties that differ given the clinical context. Nevertheless, with such challenges, we need a supportive system for imaging decision-making.

AIRI aims to address these challenges through its multimodal AI system to guide physicians in imaging decision-making. It will help physicians decide when imaging is necessary, what modality is most appropriate, and other distinctions in image orders, such as whether contrast is indicated. By leveraging patients’ clinical data, imaging appropriateness criteria, and advanced analytics, AIRI can facilitate optimized imaging orders and reduce inappropriate imaging, which would reduce financial costs, limit patient exposure to radiation, and reduce time lags for diagnosis. In addition to its usefulness for clinicians, AIRI would be a powerful tool for radiologists by reducing the occurrence of missed diagnoses through advanced image analysis. In this paper, we will explore the current role AI plays in the healthcare industry, as well as how our proposed multimodal AI system, AIRI, would build upon the existing benefits that AI provides in the medical industry. We will also explore the specific use cases of AIRI for clinicians, radiologists, and patients, and how AIRI can be implemented to address the needs of each of those groups. 

AI applications in modern healthcare: advances in diagnostics, prediction, and medical imaging applications

AI and its integration of deep learning have revolutionized medical imaging and diagnostics through its capacity to analyze complex datasets with unprecedented accuracy. Algorithms perform surprisingly well in spotting the aberrant signal hidden in the noise of the image, whether in X-rays, ultrasound (US), magnetic resonance imaging (MRI), or CT, often matching or exceeding the diagnostic performance of human experts.

Mammography was recently the focus of a landmark study appearing in Nature Medicine, by Marinovich et al. demonstrating that AI systems may be able to detect breast cancer in mammograms with significantly greater accuracy, resulting in a lower rate of both false positives and false negatives relative to that of traditional radiology [3]. The integrations of AI in breast cancer screening are far from unhelpful; they consistently help make the workflow of screen-reading and diagnostic accuracy 41.4% less (p < 0.05) when used in conjunction with a radiologist [3]. This enhancement in efficiency optimizes the screening process and effectively addresses the healthcare professional workload, enabling more strategic resource allocation. These improved workflows can save the patient and the healthcare industry money by reducing unnecessary follow-up and making much better use of the limited resources available.

The potential of AI in imaging optimization lies in improving efficiency and its ability to process massive datasets quickly and discover small patterns that can be hard for the human eye to detect. This increased analytical capability allows for more accurate diagnoses and earlier cancer detection, which has been extremely important in improving survival rates since the validation study by Marinovich et al. [3]. For example, Marinovich et al. employed their deep learning model, DeepHealth AI, that reached an area under the receiver operating characteristic curve (AUC-ROC) of 0.83 versus the radiologists’ AUC-ROC of 0.93. AUC-ROC is a performance metric that measures the model’s ability to distinguish between classes, where a score of 1.0 means perfect classification, i.e., 100% sensitivity and specificity, while 0.5 represents random chance. Although the AI performance was slightly lower, these results show its potential as a complementary tool to human expertise [3].

Most importantly, AI augments human expertise; it does not replace it. In radiological practice, AI is a second reader with optimal results, increasing cancer detection rates and helping with diagnostic decision-making [3]. This application goes beyond breast cancer diagnostics. AI has proven highly useful for other conditions, such as diabetic retinopathy, reducing the time needed to diagnose while increasing accuracy.

Predictive analytics and early disease detection

AI-powered predictive analytics transforms preventive healthcare through sophisticated patient data analysis to predict the disease’s risk and outcome. It has been incredibly effective for chronic conditions, such as diabetes, cardiovascular diseases, and sepsis. Fleuren et al. (2020) meta-analysis of the literature showed that machine-learning models can predict sepsis in hospitalized patients hours before clinical recognition, which allows for critical early interventions [4].

Current AI applications in healthcare settings demonstrate varying levels of predictive accuracy based on implementation context: emergency department (ED) models (AUC-ROC, 0.85-0.89), ICU-based implementations (AUC-ROC, 0.90-0.99), and hospital ward applications (AUC-ROC, 0.80-0.85). These performance metrics consistently surpass traditional diagnostic tools, such as the Systemic Inflammatory Response Score (SIRS) and the quick sequential organ failure assessment (qSOFA). For instance, machine-learning models achieved an AUC-ROC of 0.859 in the ED setting, significantly outperforming manual triaging methods (AUC-ROC, 0.756) [4].

While there have been measurable increases in healthcare delivery efficiency due to integrating AI into clinical workflows, such deep learning must first be achieved. Implementing AI-based prediction models has reduced the length of stay from 13.0 to 10.3 days in hospital (p < 0.01), demonstrating the significant potential for cost savings. Such systems actively utilize many data points for dynamic diagnostic information that feeds into individualized treatment architectures, such as vital signs, laboratory values, and clinical observations [4].

Operational efficiency and resource management

In healthcare, AI technologies considerably improve operational efficiency regarding resource allocation, staff scheduling, and inventory management. AI deployment has been incredibly beneficial in EDs, among the most resource-limited healthcare settings. In recent research by Ahmadzadeh et al. (2024), some successful AI algorithm deployments in ED settings focused on patient flow optimization, triage processes, and resource allocation [5].

These systems provide real-time monitoring and predictive capabilities for addressing patient wait time optimization, service flow enhancement, acuity prediction, disposition decision support, and overcrowding management, among many other areas that predictive systems could address.

AI applications in healthcare have the potential to learn from large patient datasets to help predict a patient’s medical conditions and suggest possible treatment options with ever-increasing reliability. They have proven particularly useful in areas, such as churning patient admission forecasts, optimizing bed management, alleviating operational bottlenecks, facilitating intricate diagnosis systems, and streamlining resource allocation.

Besides direct clinical uses, AI systems have been used to monitor and predict care and operations in real time, which helps providers identify the best ways to utilize their resources. Clinical applications of AI include automated staff scheduling, inventory management, and predictive maintenance planning. As a result, patient wait times have measurably declined, and care delivery has been made more efficient through digital automation [5].

Conceptualizing AIRI: design and functionality

The overuse of diagnostic imaging has been well-documented and has become a concern in health care over the last several decades. As defined in the healthcare industry, overuse is a process of care that creates more harm than good relative to its intended therapeutic or diagnostic value [6,7]. While imaging modalities like CT, MRI, and X-ray have revolutionized health care and improved health outcomes and diagnostic accuracy, overuse of these tools creates challenges for patients and the healthcare industry. These challenges include but are not limited to financial costs, excessive exposure to radiation to patients, future health complications related to excess exposure, and an increased burden on radiologists to interpret the growing volume of scans [2]. As a result, this creates a double-edged sword for healthcare workers, particularly radiologists, who are tasked with mitigating overuse amid ever-growing volumes. 

AIRI would be a transformative framework to address these challenges. Although AI has already shown much promise in various clinical applications, as described in the previous section, its potential to guide non-radiologists in ordering appropriate imaging studies remains underexplored. Non-radiologist clinicians often have received little training regarding which imaging modalities are most indicated. Clinical criteria such as the ACR Appropriateness Criteria, the Canadian C-Spine Rule, the Ottawa Ankle Rules, and the PECARN Pediatric Head and C-Spine CT Algorithm help guide clinicians; however, they are provider-specific and prone to subjectivity. A gap that AIRI has the potential to fill is maintaining consistent pre-screening of patients when considering whether a scan is indicated and will provide additional information necessary for diagnosis. AIRI would achieve this by providing evidence-based imaging recommendations to optimize the process for physicians and patients and consequently improve the workflow for radiologists. 

In short, the goal of AIRI would be to help non-radiologists determine whether a scan is essential for guiding treatment. It would also help guide their selection of the best modality in a given clinical scenario and patient preparation to ensure scans are performed under optimum conditions. As a result, AIRI would minimize radiation exposure and reduce the financial burden of imaging without compromising diagnostic accuracy. It would also facilitate the retrieval and interpretation of prior imaging studies within electronic medical records (EMRs) to prevent unnecessary repeat scans, while keeping patients better informed about clinical decisions regarding their care.

**Figure 1:**
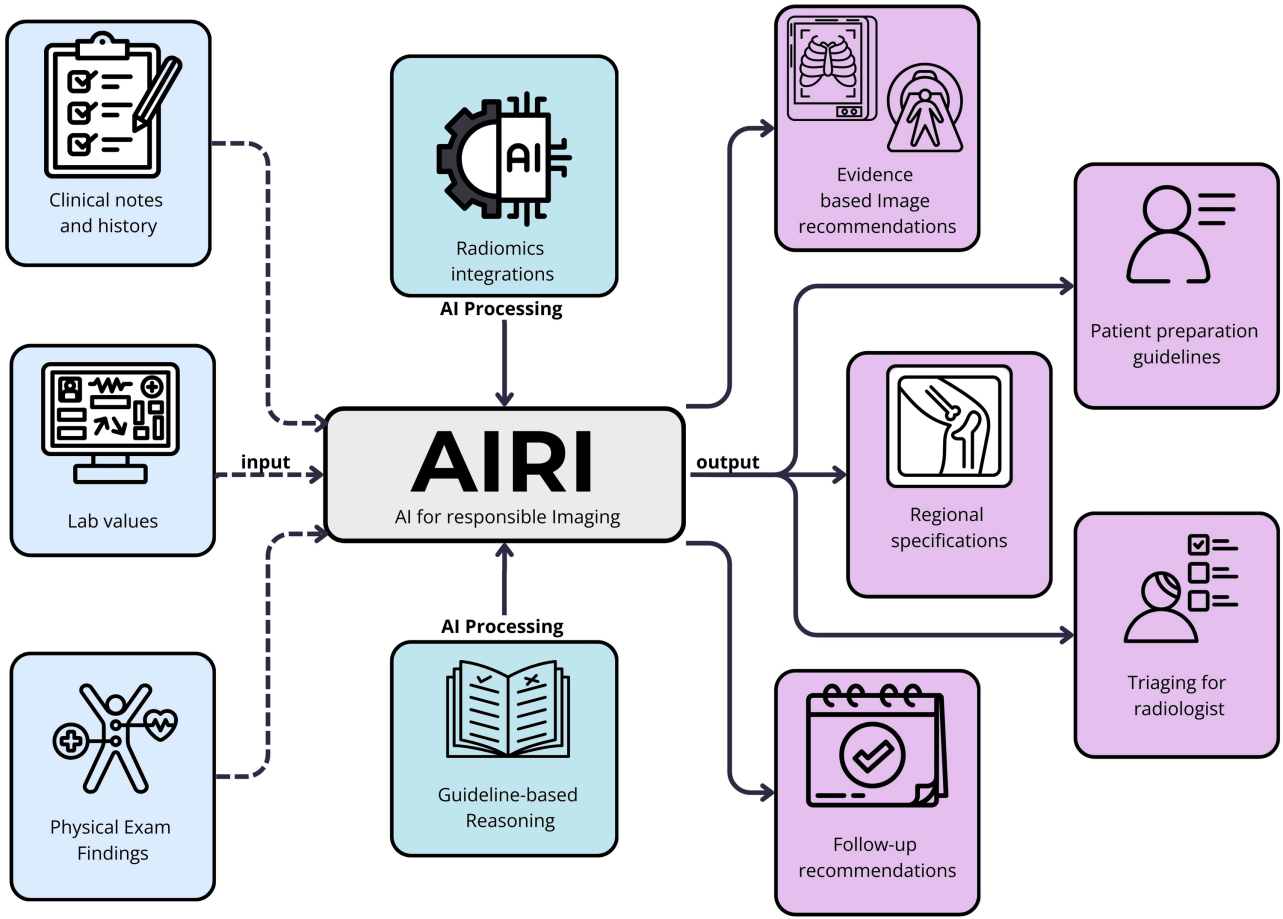
Inputs and outputs of AIRI AIRI uses clinical inputs like notes, history, lab values, and physical exam findings to provide evidence-based imaging recommendations, patient preparation guidelines, follow-up plans, triaging support, etc., to streamline the decision-making for non-radiologists and to improve workflows for radiologists. AI, artificial intelligence; AIRI: AI for responsible imaging Note: Figure created incorporating elements from ©Uniconlabs, ©Slamlabs, ©Gravisio, ©Freeicon, ©Ajan Khan from Vector Icons, ©Aratra Studio, ©Smaicons from SAM Designs, ©Ferdizzimo, and ©Anh Pham from Quynh Anh Ne via Canva.com. Permission was obtained under Canva Pro’s Content License Agreement.

The main principle on which the design of AIRI would be based is accuracy. To ensure accuracy, AIRI must be trained with evidence-based algorithms and a robust dataset of clinical case scenarios. The non-radiologist’s inputs include clinical notes, patient history, physical exam findings, and laboratory results (Figure 1). Based on these inputs and the learning algorithms and machine learning (ML) models used to train AIRI, a series of outputs would be produced to guide the non-radiologist clinician, advising the clinician on the most appropriate modality to use given the symptoms, laboratory studies, vital signs, and patient history, thus helping avoid unnecessary tests. For example, it might indicate whether a CT or US would be better for a patient with abdominal pain while recommending preparation or warning providers about possible contraindications such as allergies to contrast. The system outputs would range from imaging recommendations, guidance for patient preparation, plans for follow-up, and prioritizing imaging based on clinical urgency. With AIRI, non-radiologists can make even the most complex imaging decisions with ease. For the radiologists, AIRI’s accuracy would allow them to benefit from their streamlined workflows and reduced quantity of unneeded scans. Building AIRI would require extensive training on multimodal datasets with self-supervised learning for pattern recognition and context-specific recommendations based on a large pool of cases frequently seen in clinics, the outcomes of scans for such cases, and updated clinical guidelines, which would ensure the system continually improves through real-time cases and stays current with clinical guidelines and medical knowledge.

Furthermore, when the scans are completed, AIRI could be implemented to lighten the radiologist’s workload by suggesting preliminary interpretations and creating a framework for dictation. AIRI could also flag scans that are of low quality or disrupted by artifacts and use radiomic integration to improve the interpretation of these low-quality scans. Radiomics is an expanding field of study that is based upon the integration of quantitative data extraction for radiologic images, it was developed to recognize minute details and imaging patterns that are either challenging or impossible for the human eye to identify [8]. With the addition of radiomics, a tool for quantitative data extraction for medical images, AIRI would provide consistent and accurate imaging interpretation, including the interpretation of subtle findings that may be difficult to catch with the naked eye [8]. Beyond radiomics, AIRI could also provide templates for dictation with AI-supplemented content to enhance clarity. Additionally, regarding non-radiologists reading the report, AIRI could help standardize the radiologic vernacular to make it more understandable to the non-specialists and lessen the time spent on routine cases to free the radiologists from complex and high-priority tasks.

In short, AIRI has the potential to address overutilization, diagnostic performance, streamlining communication, and patient safety. With better training and seamless integration into clinical workflows, AIRI, as a medical tool, has the potential to play a leading role in shaping the future of medical imaging.

Clinical applications of AIRI for the clinician: examples in surgery, public health, and gastroenterology 

**Figure 2:**
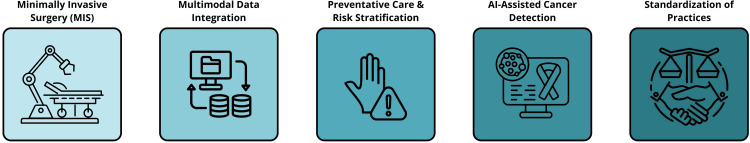
Key applications of AIRI in diagnostics and treatment Key applications of AIRI in advancing medical imaging, illustrating its role in enhancing MIS, integrating multimodal data for precision diagnostics, enabling preventative care and risk stratification, supporting AI-assisted cancer detection, and promoting the standardization of clinical practices. MIS, minimally invasive surgery; AI, artificial intelligence; AIRI, AI for responsible imaging Note: Figure created incorporating elements from ©Philipp Petzaka from Noun Project, ©Afianroc, ©Uniconlabs, ©Vectoricons, and ©VectorMine via Canva.com. Permission was obtained under Canva Pro’s Content License Agreement.

AIRI is proposed as a transformative tool in diagnostics and treatment. Imaging has become a vital tool for non-radiologist physicians, and AIRI can be crucial in enhancing responsible, accurate imaging. Multimodal data, real-time analytics, and advanced computational tools can offer a brilliant opportunity to improve patient care and clinical workflows. Figure 2 highlights key applications of AIRI in clinical practices.

Minimally invasive surgery (MIS) is an essential part of modern clinical practice that relies heavily on imaging. The integration of AIRI into MIS may assist in overcoming a chronic problem of tactile feedback loss and complications from a tactile feedback illusion. AI-based tools like augmented reality (AR) and computer vision algorithms can help with surgeons’ visual-spatial cognition. This technology overlays information onto the surgical field, allowing for targeted navigation during surgery. ML models may also play a principal part by predicting complications, optimizing surgical plans, and acting as a “second pair of eyes” to clinicians in real time [9].

One of the most exciting developments in responsible imaging is integrating multimodal data. This approach combines diverse datasets, such as fluorescence imaging, radiologic scans, genomic profiles, and pathological data. Intraoperative tools that integrate fluorescence imaging with near-infrared (NIR-II) and radiologic imaging provide surgeons with incomparable guidance during tumor resections. These tools identify molecularly distinct regions within tumors, which help inform targeted therapies, especially when combined with genomic and proteomic data. Likewise, similar AI models leveraging multimodal data in glioma surgeries showed the possibility of predicting tumor grade and ki-67 expression levels in real time to guide intraoperative decisions of resection. AI further closes the space between imaging and molecular pathology. Fluorescence imaging, for example, indicates metabolic activity and blood perfusion, whereby pathological analyses demonstrate tumor type and grade. Together, these insights provide a holistic view of tumor heterogeneity, enabling tailored treatment strategies in real time [9].

Beyond the operating room, preventative guidelines and tools are essential for improving public health outcomes. The need to make these as sensitive and specific is an ongoing battle for research. AI has been and can continue to be a potent tool to increase both the accuracy and speed of detection. AI can integrate electronic health records, imaging, and genomic data, allowing us to polish our predictive models further. This integrative view can yield insights that are tedious and will enable the detection of early signs of a worsening disease or therapeutic resistance. For instance, AIRI tools may enable patient risk stratification, allowing clinicians to prioritize immediate follow-up or specific interventions for higher-risk patients. We are already seeing this in ERs for triage. This ability could also enable clinicians to see more patients daily without detracting from the caliber of care [10].

AI has particularly shown great strides in the early detection of diseases like colorectal cancer (CRC). Screening for CRC is dependent on identifying precancerous polyps; these subtle lesions are often missed during standard colonoscopies. AI-assisted colonoscopy systems can revolutionize real-time polyp detection, such as computer-aided detection (CADe) and diagnosis (CADx). These systems analyze video feeds during colonoscopy procedures, identifying adenomas with upwards of 91% sensitivity and specificity. Convolutional neural networks (CNNs), a specialized form of AI, highlight suspicious regions in real time, reducing the risk of missed diagnoses and improving adenoma detection rates (ADRs) by 30-50% [10]. The minimization of human error and the precision of AI reduces the number of redundant procedures as patients no longer have to go through unnecessary procedures, which in turn reduces overall healthcare costs.

Beyond cancer, AI is finding a role in preventative care across various domains. Algorithms trained on different datasets can detect early signals of chronic diseases like diabetes and cardiovascular disease. AI can provide clinicians with actionable insights to prevent disease progression and medication choices by analyzing electronic health records, imaging data, and even wearable device metrics. This integration is already visible in EMR systems like Epic, where quality checks for patients on medications such as statins or alerts for routine diabetic ophthalmology screenings self-populate in the chart. This feature can be further elevated by AIRI, which can mark patients as high-alert or attention-needed labels when preventive screenings for mammograms or colonoscopies, for example, are recommended by radiology as abnormal or in need of further testing. In these scenarios, prevention is not just about detecting a condition but about predicting and reducing the chances of missed diagnoses for physicians overwhelmed by patient load.

One of AIRI’s most significant benefits will be aligning and standardizing physician practice. That combination, however, presents challenges, as more and more healthcare providers train in various backgrounds and roles and as the push for more autonomy grows. By integrating evidence-based principles into processes, AIRI can help all clinicians adhere to the same standards when ordering imaging, regardless of experience level. AI-powered education platforms could bridge gaps in training by catering to learning modules based on the provider’s need. AI can also analyze data from multiple providers to identify patterns of practice variation. This information obtained by AIRI may help organizations focus their training or adjust processes to address gaps. Standardization may also lead to better outcomes for patients. Standardization would improve patient outcomes and decrease the legal risks associated with variability in care. However, it is also essential that systems like AIRI be set up to respect the nuances of clinical judgment and avoid over-standardization and overly rigid pathways that might preclude the creativity or adaptability of individual physicians in unique or unusual clinical situations.

Clinical applications of AIRI for radiologists

**Figure 3:**
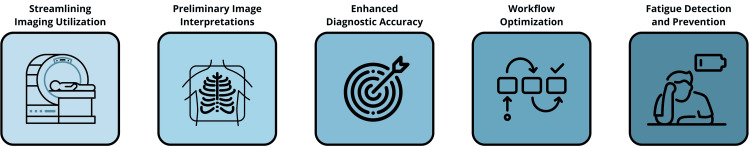
Key applications of AIRI in radiology practice Key clinical applications of AIRI for radiologists, emphasizing its role in streamlining imaging utilization, providing preliminary image interpretations, enhancing diagnostic accuracy, optimizing workflows, and implementing fatigue detection and prevention to improve efficiency and ensure high-quality care in medical imaging. AIRI, artificial intelligence for responsible imaging Note: Figure created incorporating elements from ©Pat Librojo from sparklestroke, ©Victoruler from Noun Project, ©Setyoartech Gallery, ©Darwoi Mulya, and ©Sir.Vector via Canva.com. Permissions were obtained under Canva Pro’s Content License Agreement.

Overusing diagnostic imaging has placed a significant burden on radiologists responsible for reading, interpreting, and dictating a large volume of images. A strategic way to mitigate the issue of imaging overutilization is to streamline the number of patients being scanned by ensuring that most, if not all, of the scans being performed are clinically indicated and based on the algorithmic likelihood that imaging would provide additional information. As described in previous sections, AIRI can provide this level of streamlining by guiding the non-radiologist toward ordering specific scans only when clinically necessary. However, even with such a reduction in unnecessary imaging, the radiologist will still have many scans to interpret. Figure 3 illustrates the key clinical applications of AIRI for radiologists, and how AIRI would benefit the radiologist, through its ability to provide preliminary interpretations, improve diagnostic accuracy, etc.

To further optimize the usefulness of AIRI, it would be beneficial for the tool to provide preliminary interpretations for low-priority images or frequently seen cases. Preliminary interpretations could be achieved by integrating radiomics into AIRI for image interpretation. Using radiomics would allow the radiologist to focus on higher priority or more complex cases, but it would also improve diagnostic accuracy and help standardize image interpretations [8]. Furthermore, the integration of radiomics into AIRI’s framework has the potential to pick up on minuscule abnormalities that may be challenging to visualize by the naked eye [8]. For example, AIRI could help radiologists achieve faster turnaround times for lower-priority imaging studies like fracture scans without missing subtle findings. AIRI could also identify low-quality scans or images distorted by artifacts and utilize its radiomics integration to pick up information distorted by artifacts created by movement, hardware, or other sources to reduce the need for repeat scans in such scenarios. 

Moreover, as AIRI develops and integrates into the healthcare industry, it could also be used to optimize workflow by providing AI fatigue detection to prevent radiologist burnout. AIRI could be trained to incorporate triaging based on clinical urgency and the radiologist’s productivity level. AIRI is a step forward in reducing radiologists’ fatigue, improving diagnostic accuracy, and optimizing workflows to enhance the overall quality and efficiency of medical imaging.

Clinical applications of AIRI for patients

**Figure 4:**
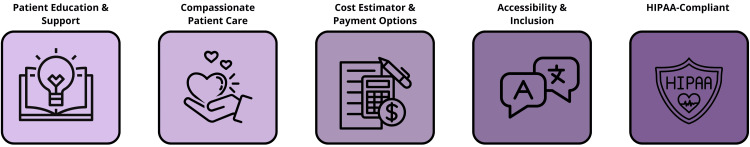
Key applications of AIRI in patient-centered care Key clinical applications of AIRI for patients include patient education and support, compassionate patient care, cost estimation and payment options, accessibility and inclusion, and adherence to HIPAA compliance standards to enhance the patient experience and improve healthcare accessibility. HIPAA, Health Insurance Portability and Accountability Act; AIRI, artificial intelligence for responsible imaging Note: Figure created incorporating elements from ©Chattapat, ©Creativemahira, ©Amethyststudio, ©Fran Couto from Noun Project, and ©Glyphium Artistry from Aimen Abbas via Canva.com. Permission was obtained under Canva Pro’s Content License Agreement.

AIRI is beneficial not only for healthcare providers but is also envisioned to be an informative and interactive tool for patients. While AIRI may help healthcare professionals make decisions while ordering or reading images for their patients, it can also help patients similarly. It can help patients understand the imaging indications, explain the findings in imaging studies, and offer recommendations for further management. Patients can better understand their diagnosis and health management in an outpatient setting by incorporating patient-centered tools into AIRI. Figure 4 illustrates the interactive capabilities of AIRI for patients. The benefit of AIRI for patients is its ability to keep patients informed and educated, allowing them to make informed decisions about the direction of their care.

AIRI has the potential to be a supportive tool for patients. The future directions of this AI tool would incorporate a conversational chatbot for patients to use, allowing them to stay updated and informed about their care and the decisions typically made on their behalf. The AIRI chatbot would be trained to answer patients’ questions by pulling from their medical records to keep the design HIPAA compliant. Furthermore, patients often have curiosities about the difference between the various imaging modalities, so having a chatbot tool to describe why a specific modality was ordered could help resolve any confusion. Furthermore, as discussed in previous sections, AIRI can tell what to expect during various imaging procedures and help prepare the patient. For example, if they are supposed to fast before a procedure, it is essential that the patient also understand why fasting is needed to keep them informed about their care and to improve compliance.

Additionally, patients often experience anxiety and fear during scans. For example, many patients experience claustrophobia, motion sickness, and other related anxieties during CTs or MRIs. All patients should be aware of this risk before receiving imaging and be properly informed before consenting [11]. AIRI’s conversational component could address these anxieties by not only being friendly, empathetic, and understanding when addressing their concerns but also providing the patient with medical or counseling options to address such anxieties before being imaged. A study by Liszio et al. proposed the use of VR headsets during imaging procedures as a tool to address anxieties related to being scanned [12]. As tools like this become more well-researched and widely available, AIRI could make broader and more personalized suggestions to address such cases [12]. 

AIRI, with the help of healthcare providers, could also improve the patient experience by suggesting next steps based on imaging results. For example, AIRI could provide follow-up recommendations with an orthopedic specialist or a physical therapist for a patient who is diagnosed with a degenerative joint disease following an imaging study. Depending on that particular patient’s history, AIRI would provide recommendations for the patient to follow up. 

Furthermore, one of the main barriers to accessing imaging is the absence of price disclosure. While a study by Paul et al. discovered that the radiology industry is relatively more transparent in providing patients with cost estimates compared to other specialties such as general surgery, it interestingly found that prices often vary significantly between different institutions ranging from five- to 10-fold differences in price-meaning that depending on where a patient receives a scan, they could save themself hundreds to thousands of dollars [13].

This is important because AIRI could assist patients by comparing prices and directing them to nearby scanning locations with lower rates. While the study by Paul et al. reported that within the field of radiology, about 78-79% of upper-tier academic hospitals and private practices were transparent about their imaging prices, which is higher than in other healthcare fields, it is essential to note that there is still room for improvement [13].

An additional feature in AIRI that can solve this problem would be a cost estimator based on data from previous patients with similar cases. It could give patients a clear structure of the imaging costs, including the expenses, insurance coverage, deductibles, and out-of-pocket costs. This function would provide patients with a price breakdown of their imaging procedure. Financial difficulty is another barrier that can hinder patients’ compliance with imaging recommendations. AIRI would offer patients facing financial barriers options for available payment plans to potentially alleviate some of the economic burden.

Another barrier to patient care that we feel is important to address is accessibility. Developing accessibility features for AIRI would include text-to-speech, closed captioning, and easy-to-use interactive interfaces to ensure the AI tool is accessible. Integrating these accessibility features would be especially useful for patients with visual, auditory, or cognitive impairments. A study by Kuenburg et al. suggests a need for medical professionals to be properly trained to competently and skillfully interact with patients who have such disabilities, specifically deaf patients as in this study [14]. AIRI would improve these types of interactions with patients with special needs, allowing the physician to effectively communicate with their patient in a personalized manner. Additionally, AIRI should be available in multiple languages to ensure broader patient access. Imaging reports are often complex and written in medical jargon, making them difficult for patients to understand. With AIRI, patients can simplify and interpret their imaging findings while receiving answers to any questions they may have. This would make AIRI a valuable educational tool, helping patients stay informed about their health.

AIRI has many capabilities and can catalyze technological advancements in radiology regarding patient forward thinking and care. However, it must be emphasized that AIRI is intended to complement radiology and assist those who work in the field. As such, the purpose of AIRI is to assist, not replace, human interaction between the patient and the healthcare providers.

Conclusion and future directions

The intersection of radiology and AI is still at an early stage, yet it is already transforming the field of medical imaging. AIRI would fit seamlessly into the current field of medical innovation, as it can advance the responsible use of medical imaging by assisting clinicians in making more accurate decisions and aiding radiologists in interpretation. Our vision for AIRI has the potential to move the field of radiology toward a new era that is based on efficiency, patient safety, and cost-effectiveness.

AIRI’s innovative aspects include standardizing imaging recommendations across various clinical situations based on clinical inputs, such as vital signs, laboratory results, physical exam findings, and history. With the proposed multimodal data integration, AIRI could create a space for more personalized and accurate medical imaging recommendations. Ensuring patients are not subject to unnecessary or incorrect imaging utilization. 

Further advancements in AIRI’s development are crucial to ensure continued self-learning and training of the AI tool, as guidelines and indications are subject to change with additional research. In the future, developing AIRI’s learning algorithms with larger and more robust datasets will be crucial to improve its performance in complicated clinical presentations. 

While AIRI is envisioned to enhance the field of radiology and assist clinicians, it is not a replacement for human expertise. Instead, it is a tool to aid the decision-making process. Ultimately, the vision for AIRI is to set a new standard in medical imaging that balances precision, efficiency, and patient safety. 

## References

[1] Moor M, Banerjee O, Abad ZS, Krumholz HM, Leskovec J, Topol EJ, Rajpurkar P (2023). Foundation models for generalist medical artificial intelligence. Nature.

[2] Oren O, Kebebew E, Ioannidis JP (2019). Curbing unnecessary and wasted diagnostic imaging. JAMA.

[3] Marinovich ML, Wylie E, Lotter W (2023). Artificial intelligence (AI) for breast cancer screening: BreastScreen population-based cohort study of cancer detection. EBioMedicine.

[4] Fleuren LM, Klausch TL, Zwager CL (2020). Machine learning for the prediction of sepsis: a systematic review and meta-analysis of diagnostic test accuracy. Intensive Care Med.

[5] Ahmadzadeh B, Patey C, Hurley O (2024). Applications of artificial intelligence in emergency departments to improve wait times: protocol for an integrative living review. JMIR Res Protoc.

[6] Kwee RM, Toxopeus R, Kwee TC (2024). Imaging overuse in the emergency department: the view of radiologists and emergency physicians. Eur J Radiol.

[7] Hendee WR, Becker GJ, Borgstede JP (2010). Addressing overutilization in medical imaging. Radiology.

[8] Mayerhoefer ME, Materka A, Langs G, Häggström I, Szczypiński P, Gibbs P, Cook G (2020). Introduction to radiomics. J Nucl Med.

[9] Cheng H, Xu H, Peng B, Huang X, Hu Y, Zheng C, Zhang Z (2024). Illuminating the future of precision cancer surgery with fluorescence imaging and artificial intelligence convergence. NPJ Precis Oncol.

[10] Mitsala A, Tsalikidis C, Pitiakoudis M, Simopoulos C, Tsaroucha AK (2021). Artificial intelligence in colorectal cancer screening, diagnosis, and treatment. A new era. Curr Oncol.

[11] Nguyen XV, Tahir S, Bresnahan BW (2020). Prevalence and financial impact of claustrophobia, anxiety, patient motion, and other patient events in magnetic resonance imaging. Top Magn Reson Imaging.

[12] Liszio S, Basu O, Masuch M (2020). A universe inside the MRI scanner: an in-bore virtual reality game for children to reduce anxiety and stress. Proceedings of the Annual Symposium on Computer-Human Interaction in Play.

[13] Paul AB, Oklu R, Saini S, Prabhakar AM (2015). How much is that head CT? Price transparency and variability in radiology. J Am Coll Radiol.

[14] Kuenburg A, Fellinger P, Fellinger J (2016). Health care access among deaf people. J Deaf Stud Deaf Educ.

